# Structural brain changes, cerebral oxygenation, and cognitive function in hemodialysis patients: a cross-sectional study

**DOI:** 10.3389/fmed.2026.1885904

**Published:** 2026-07-10

**Authors:** Martin Michna, Romana Burgetova, Karin Kremenova, Andrea Burgetova, Jan Krasensky, Jiri Lukavsky, Barbora Szonowska, Ivan Rychlik, Hana Malikova, Jan Malik

**Affiliations:** 1 Department of Radiology and Nuclear Medicine, Third Faculty of Medicine, Charles University and University Hospital Kralovske Vinohrady, Prague, Czechia; 2 Third Department of Internal Medicine, General University Hospital in Prague, First Faculty of Medicine, Charles University, Prague, Czechia; 3 Department of Radiology, First Faculty of Medicine, Charles University and General University Hospital in Prague, Prague, Czechia; 4 Faculty of Arts, Charles University, Prague, Czechia; 5 Fresenius Medical Care, Kralovske Vinohrady, Prague, Czechia; 6 Department of Internal Medicine, Third Faculty of Medicine, Charles University and University Hospital Kralovske Vinohrady, Prague, Czechia

**Keywords:** atrophy, CKD, cognition, hemodialysis, oxygenation

## Abstract

**Background:**

Cognitive impairment is frequent in end-stage kidney disease (ESKD) patients, who also have brain structural changes and reduced regional cerebral oxygenation (rSO2). However, the relationship between these parameters remains insufficiently explored. The primary aim of this study was to compare cognition, brain structural changes and cerebral oxygenation between hemodialysis (HD) patients and healthy controls. The secondary aim was to assess the relationship between cerebral oxygenation, cognitive performance and brain morphometric parameters in HD patients.

**Methods:**

In this cross-sectional study, we enrolled 44 HD patients (24 males, 20 females; mean age 72.5 ± 17.5 years) and 24 healthy controls of similar age. All patients had received HD for more than three months. Cognitive function was assessed using the Montreal Cognitive Assessment (MoCA). Frontal lobe oxygenation was measured with the INVOS 5100C Oximetry system. Brain morphology was evaluated using MRI on a Magnetom Sola 1.5 T scanner, processed with MorphoBox software.

**Results:**

The HD group showed lower rSO2 (*p* < 0.001), worse cognitive scores (p < 0.001), and significant brain structural changes including reduced white (*p* = 0.005) and grey (*p* = 0.002) matter volumes, more extensive white matter hyperintensities (*p* = 0.04), microbleeds (*p* = 0.001), and lacunar infarcts (*p* = 0.003). MoCA and rSO2 values were positively correlated in controls (Spearman rho = 0.411, *p* = 0.046), but not in HD patients. In the HD group, MoCA scores significantly correlated with brain structural parameters (*p* < 0.05).

**Conclusion:**

HD patients had impaired cognition, lower cerebral oxygenation, and marked structural brain changes. However, regional frontal cerebral hypoxia was not associated with cognitive decline or brain structural changes in HD patients.

## Graphical abstract

**Figure:**
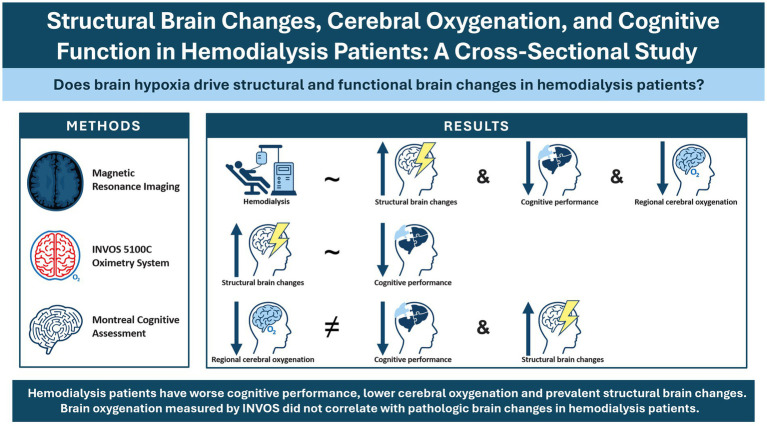
Graphical abstract

## Highlights

- Hemodialysis patients have more advanced brain morphological changes and these are related to cognitive decline.
- Regional cerebral oxygenation is reduced in HD patients, but it was not significantly associated with cognitive impairment or brain structural changes.
- Lower baseline cerebral oxygenation may be weaker mechanism than was supposed

## Introduction

Chronic kidney disease (CKD) increases the risk of cognitive impairment (1, 2). The prevalence of dementia in end-stage kidney disease (ESKD) is approximately three times higher than that in the general population (3). In addition, ESKD patients exhibit brain morphological changes, such as accelerated brain atrophy (4, 5) and white matter damage (6, 7).

The aetiology of cognitive deficit is multifactorial and involves also mechanisms that lead to CKD, such as diabetes mellitus and arterial hypertension (8). Several metabolic factors, including uremic toxins, altered plasma osmolarity (9–12), chronic low-grade inflammation (13) and endocrine changes (14, 15) also influence the development of cognitive deficit. Hemodynamic factors, including the frequent occurrence of heart failure (16), loss of arterial wall elasticity (17) and impairment of cerebral blood flow autoregulation (18), further contribute to cognitive decline. Lastly, factors related directly to the hemodialysis (HD) procedure, such as rapid changes in fluid and hemodynamic instability during HD sessions, may also impact cognitive function (19).

The presence of ESKD, as well as HD treatment itself, are associated with brain structural changes and cognitive decline beyond what is observed in the general population (20). A dominant factor contributing to these changes may be chronic cerebral hypoxia (21–23). Multiple pathophysiological mechanisms in ESKD converge toward reduced cerebral oxygenation. Uremic toxins, chronic low-grade inflammation, and cerebrovascular disease promote endothelial dysfunction, which directly impairs cerebral microvascular perfusion and oxygen delivery (24, 25). These same factors also disrupt the blood–brain barrier (26–28). Patients with ESKD display approximately twofold higher blood–brain barrier permeability compared to healthy controls, and this correlates inversely with cognitive performance (29). Blood–brain barrier disruption impairs neurovascular coupling and local perfusion regulators (30). Cerebral perfusion is further compromised during HD sessions, when cerebral blood flow declines by approximately 10% (31). These repetitive intradialytic episodes of cerebral hypoperfusion may contribute to progressive brain injury (32). Once the blood–brain barrier is compromised, neurotoxic substances may enter the brain parenchyma, promoting further neuroinflammation and neuronal injury (33).

The near-infrared spectroscopy (NIRS) is used for a non-invasive brain oxygenation assessment. It uses infrared light in the 700–1,000 nm wavelength range. In this spectrum, light can penetrate several centimeters into biological tissues, enabling continuous monitoring of hemoglobin oxygen saturation in cerebral or peripheral regions. However, its use is limited by relatively shallow penetration depth (<2.5 cm).

In general, several mechanisms of brain morphological changes and cognitive decline associated with chronic HD therapy are described. Unfortunately, the links between brain morphological changes, brain oxygenation and cognitive function have not been studied in detail. The primary aim of the study was to compare brain structural, functional, and oxygenation parameters between hemodialysis patients and healthy controls. The secondary aim was to assess the relationship between cerebral oxygenation, cognitive performance and brain morphometric parameters in HD patients.

## Materials and methods

The study was approved by the local ethics committee of the University Hospital Kralovske Vinohrady and of the Third Medical Faculty of Charles University (EK-VP/56/0/2021). The study was conducted in compliance with the Helsinki Declaration. All the participants in the study provided informed signed consent prior to their enrolment.

### Patient selection

We conducted a single-center cross-sectional study involving adult subjects aged 18 years or older who were undergoing treatment by chronic HD therapy for more than 3 months and were willing to participate in the study. The HD patients underwent a standard hemodialysis regimen of three sessions per week. Each session typically lasted 4–5 h, consistent with the standard treatment protocol for patients with ESKD. Data were collected between November 2021 and March 2024.

The exclusion criteria were as follows: decompensated chronic heart failure, history of cerebrovascular accidents, known neurodegenerative disorder or other chronic neurological disorders, chronic pulmonary diseases such as chronic obstructive pulmonary disease and pulmonary fibrosis, contraindications for MRI (including MRI non-compatible implanted cardiac pacemakers or defibrillators, retained leads post-explantation of pacemakers or defibrillators, electronic implants, metallic foreign bodies made of materials other than clearly non-magnetic metals intracranially or intraorbitally), and claustrophobia.

The control group consisted of healthy volunteers with similar age and sex to the HD patients. We used frequency matching to ensure that the overall distribution of age (by decades) and sex was similar between the groups, without matching each participant individually. The healthy controls were recruited from among employees of our institution, who were invited via a mass email and voluntarily agreed to participate in the study. Eligibility criteria of the control group included the absence of chronic kidney disease, chronic pulmonary diseases, history of stroke and neurological or cognitive disorders.

### Cognitive functions assessment, MRI examinations and cerebral tissue saturation measurement

The examinations were conducted at least 24 h after the preceding HD session to exclude the possible acute hemodynamic effects of HD.

Cognitive function was assessed and quantified using the Czech version of the MoCA test (34). This validated screening instrument is designed to evaluate various cognitive domains, including memory, attention, language, visuospatial abilities and executive function. Data collection and evaluation were performed by a qualified professional (MM) with substantial expertise in this methodology and who had received relevant training. The MoCA test was consistently administered by the same examiner to ensure unbiased and comparable results. In accordance with standard MoCA administration guidelines, an education correction was applied to the total score where appropriate (one point was added to the total MoCA score for participants with ≤12 years of formal education).

Brain morphological changes were examined based on MRI scans conducted using a 1.5 T MR scanner (Magnetom Sola, Siemens Healthineers, Erlangen, Germany). The imaging protocol consisted of the following sequences: T2 BLADE, diffusion-weighted imaging (DWI) with b-values of 0 and 800, along with an ADC map, susceptibility-weighted imaging (SWI), T1-weighted MPRAGE and T2 SPACE DARK FLUID. The technical parameters of all the MRI sequences are provided in Table 1. No gadolinium contrast agent was administered.

**Table 1:** Acquisition parameters of magnetic resonance imaging.

Sequence	TR (ms)	TE (ms)	TI (ms)	Slice thickness (mm)	Layer gap (mm)	FOV (mm)	In-plane resolution (mm)
T2 BLADE	3,590	106	–	4	1	228	0.5 × 0.5
T2 SPACE DARK FLUID	7,600	431	2,400	1	0	250	0.5 × 0.5
DWI	5,870	60	–	5	1.5	230	1.4 × 1.4
SWI	48	40	–	3	0	230	0.9 × 0.9
T1 MPRAGE	2,200	3	–	1	0	250	1 × 1

DWI–diffusion-weighted imaging; SWI–susceptibility-weighted imaging; TR–repetition time; TE–echo time; TI–inversion time.

We assessed the following morphological changes and pathological findings: the presence and number of microbleeds, the presence and number of lacunar infarcts, brain volumetry (which included total intracranial volume, thalamic volume, cortical grey matter volume, brain volume, amygdala volume, hippocampal volume and white matter volume), and the burden of white matter hyperintensities (WMH).

Volumetric analysis of brain structures was conducted using a fully automated pipeline implemented in the MorphoBox software (Advanced Clinical Imaging Technology Group, Lausanne, Switzerland) (35). High-resolution 3D T1-weighted MPRAGE images served as input for the analysis. The processing pipeline included skull stripping, tissue classification, and atlas-based segmentation. MorphoBox uses probabilistic brain atlases and intensity models to segment the brain into gray matter, white matter, and cerebrospinal fluid compartments. It also provides estimates of regional brain volumes based on anatomical structures defined by the Neuromorphometrics atlas. Total intracranial volume was computed to allow normalization of individual brain volumes. The output included volumes of key brain regions such as total brain, cortical gray matter and subcortical structures.

The burden of WMH was assessed by *LST-LPA* (Lesion Segmentation Tool – Lesion Prediction Algorithm), which runs within the SPM (Statistical Parametric Mapping) framework configured within MATLAB [MATLAB version: 9.13.0 (R2022b), Natick, Massachusetts: The MathWorks Inc.; 2022]. LPA is a fully automated method based on a logistic regression model trained on a large sample of patients with WMH. The algorithm uses 3D FLAIR images as input and optionally co-registers them to the corresponding T1-weighted MPRAGE images to improve spatial accuracy. It then generates a lesion probability map, which is thresholded to produce a binary lesion mask. From this mask, the total white matter volume was automatically calculated for each subject. Lesion volumes were normalized to total intracranial volume to account for inter-individual differences in brain size. All segmentation outputs underwent visual quality control to ensure anatomical plausibility and accuracy of lesion delineation.

The presence and number of microbleeds were evaluated from SWI sequences by two observers (MM and RB). Similarly, lacunar infarcts were assessed from T2 BLADE and T2 SPACE DARK FLUID sequences by the same observers.

rSO_2_ was measured non-invasively using the INVOS 5100C Oximetry system (Medtronic, Essex, UK). The procedure was performed in a quiet environment with the patient sitting in a slightly reclined position. INVOS electrodes were placed on the patient’s forehead, covering both frontal lobes. Brain oxygenation was measured continuously for 5 min. Every 30 s, the oxygenation values of the right and left frontal lobes were recorded. After the measurement, the values from each side were averaged to obtain the final rSO₂ value for each frontal lobe. These averaged values were used for further analysis.

We also collected data on patients’ age, BMI, history of smoking, duration of HD, comorbidities (including arterial hypertension, diabetes mellitus, ischemic heart disease, and chronic compensated heart failure), hemoglobin levels, type of vascular access, and the use of anxiolytics, antidepressants, and anticholinergic medications. Data on patients’ dialysis regimens, such as dialysate composition, quality, and temperature, were not collected.

We correlated comorbidities and the type of vascular access with the results of the MoCA, rSO_2_ measured by the INVOS system and volumetric brain morphometry.

### Statistical methods

For the group comparisons, statistical analyses were conducted using STATISTICA software (StatSoft, USA) and R (R Core Team, 2023). Before the group comparison analyses, the distribution of the collected data was tested for normality by applying the Lilliefors test, with all variables showing non-normal distribution. Consequently, the descriptive statistics are presented as medians and quartile ranges. HD patients versus the controls were compared by applying the Mann–Whitney *U*-test, Fischer exact test and Chi-squared test. The microbleeds and lacunar infarcts were evaluated for statistical significance by applying the Chi-square test. The relationships between the variables were analyzed by applying the Spearman rank test due to the non-normal distribution of the data. For the visualization of the relationship between rSO_2_ and MoCA, we applied the robust regression method using an M estimator, fitted with iterated reweighted least squares (IWLS) to limit the impact of outliers. We performed the sensitivity analysis by the comparisons (correlations) of MoCA vs. rSO_2_ first with including all data and then again with outlier data excluded.

A Wilcoxon signed-rank test was applied to compare the oxygenation levels on the arterio-venous fistula (AVF) side and the contralateral side. Statistical tests with a *p*-value <0.05 were considered statistically significant.

We have performed a inter-rater reliability assessment for the two manually rated imaging markers (cerebral microbleeds and lacunar infarcts). Both raters (MM and RB) independently evaluated all scans, and agreement was quantified using weighted Cohen’s kappa.

Bonferroni correction was applied to confirmatory analyses involving multiple simultaneous comparisons of the same type of variable: between-group comparisons of demographic and clinical characteristics (Table 2; corrected threshold 0.05/12 = 0.0042), brain volumetric measures between groups (Table 3; corrected threshold 0.05/7 = 0.0071), and correlations of MoCA and rSO₂ with morphometric parameters (Table 4; corrected threshold 0.05/7 = 0.0071). Other parameters in Table 3, including rSO₂, MoCA, microbleeds, lacunar infarcts and T2 lesion volume, represent physiologically distinct outcome domains and were analyzed without correction. Additional exploratory analyses, each addressing a single hypothesis between unrelated variables, were also reported without correction.

**Table 2:** Characteristics of hemodialysis patient and healthy controls.

	HD patients	Healthy controls	*P*
Number of patients	44	24	–
Male	24	14	0.8
Female	20	10	0.8
Age (years)	72.5 ± 17.5	67.6 ± 11.8	0.1
BMI (kg/m^2^)	26.5 ± 6.2	26.4 ± 3.3	0.1
Vintage of HD (months)	40.5 ± 58	–	–
Permcath (%)	23	–	–
Arteriovenous fistula (%)	77	–	–
Hemoglobin (g/L)	107.8 ± 14.9	–	–
Arterial hypertension (%)	100	46	**<0.001**
Diabetes mellitus (%)	41	17	0.06
Ischemic heart disease (%)	36	4	**0.003**
Heart failure (%)	16	0	0.046
History of smoking (%)	45	17	0.02
Use of antidepressants (%)	27	4	0.02
Use of anticholinergics (%)	14	8	0.7
Use of anxiolytics (%)	9	0	0.3

HD, hemodialysis. Comorbidities, smoking history, medication use, and vascular access type are expressed as percentages. Age, BMI, hemoglobin, and dialysis vintage are shown as mean ± SD. The *p*-values in bold are statistically significant with Bonferroni-corrected threshold 0.05/12 = 0.0042.

**Table 3:** Comparison of the results between HD patients and healthy controls.

	HD patients	Healthy controls	*P*
rSO_2_ (%)	55.8 (12.0)	65.3 (11.8)	**<0.001**
MoCA (points)	24 (5)	27 (3)	**<0.001**
Mean thalamic volume (cm^3^)	6.3 (1.1)	6.9 (1.2)	0.03
Cortical grey matter volume (cm^3^)	429 (114)	444 (92)	**0.002**
Brain volume (cm^3^)	979 (199)	1,029 (210)	**0.005**
Total intracranial volume (cm^3^)	1,438 (152)	1,522 (165)	0.128
Right amygdala volume (cm^3^)	1.09 (0.21)	1.16 (0.21)	0.27
Left amygdala volume (cm^3^)	1.05 (0.18)	1.15 (0.19)	0.047
Right hippocampal volume (cm^3^)	3.13 (0.55)	3.37 (0.49)	0.09
Left hippocampal volume (cm^3^)	3.19 (0.57)	3.47 (0.48)	0.06
White matter volume (cm^3^)	360 (98)	410 (97)	**0.005**
White matter hyperintensities (cm^3^)	5.6 (14)	2.7 (8.3)	0.04
Cerebral microbleeds (%)	43.2	4.2	**0.001**
Lacunar infarcts (%)	29.5	0	**0.003**

HD, hemodialysis; rSO_2 -_ regional cerebral oxygen saturation; MoCA–Montreal Cognitive Assessment. Brain volumetric measures are reported as absolute values (cm^3^) and were not normalized to total intracranial volume. Data are presented as median and interquartile range. The results in bold are statistically significant. The Bonferroni correction was applied exclusively to brain volumetric measures, whereas no correction was performed for other data. Bonferroni-corrected threshold of 0.05/7 = 0.0071.

**Table 4:** Relationship of MoCA and rSO_2_ to other variables in HD patients.

	MoCA		rSO_2_	
	rho	*P*	rho	*P*
Age	**−0.60**	**0.001**	−0.04	0.818
Mean amygdala volume	0.33	0.033	−0.05	0.735
Mean hippocampal volume	**0.41**	**0.006**	0.03	0.865
Volume of cortical grey matter	**0.41**	**0.006**	0.09	0.578
Volume of white matter hyperintensities	−0.36	0.0217	−0.25	0.101
Brain volume	**0.61**	**<0.001**	0.14	0.389
Volume of white matter	**0.48**	**0.001**	0.15	0.354

MoCA - Montreal Cognitive Assessment; rSO_2_ - regional cerebral oxygen saturation. The results in bold are statistically significant with Bonferroni-corrected threshold 0.05/7 = 0.0071.

Power analysis: We performed a power analysis to estimate the sample size required in the comparison between hemodialysis patients and controls. It was based on the WMH volume analysis and rSO2. Based on preliminary 5 pilot patients and 5 non-dialysis controls (not included in this study), we assumed a mean difference of WMH 5% and mean difference in rSO2 15 percent points. Using a two-tailed alpha 0.05, statistical power of 0.80 and assumed between group correlation 0.8, the analysis indicated that a minimum of 25 patients would be required.

## Results

### Patient selection data

We enrolled 44 HD patients (24 males, 20 females; mean age 72.5 ± 17.5 years) and 24 healthy controls (14 males, 10 females; mean age 67.6 ± 11.8 years, *p* = 0.1). No statistically significant differences were observed between the groups in terms of age, gender, or BMI. The characteristics of the HD group and between-group comparisons are summarized in Table 2.

The HD patients had significantly lower brain oxygenation than healthy controls (55.8 ± 12.0% vs. 65.3 ± 11.8%, *p* < 0.001) and worse cognitive performance (24 ± 5 points, vs. 27 ± 3 points, *p* < 0.001) (Table 3). Likewise, the HD patients had significantly lower whole brain volume (979 ± 199 vs. 1,029 ± 210 cm^3^, *p* = 0.005), cortical grey matter (429 ± 114 vs. 444 ± 92 cm3, *p* = 0.002), white matter volume (360 ± 98 vs. 410 ± 97 mL, *p* = 0.005) and non-significantly mean thalamic volume (6.3 ± 1.1 vs. 6.9 ± 1.2 cm^3^, *p* = 0.03, non-significant after Bonferroni correction, threshold 0.0071) (Table 3). We found significantly more WMH in the HD patients (total WMH volume 5.6 ± 14 vs. 2.7 ± 8.3 cm3, *p* = 0.04), as well as a significantly more frequent cerebral microbleeds (43.2 vs. 4.2% of patients/controls, *p* = 0.001) and lacunar infarcts (29.5 vs. 0% of patients/controls, *p* = 0.003) (Table 3). The inter-rater agreement was excellent for both cerebral microbleeds and lacunar infarcts, with a weighted *κ* of 1.00 for each. Differences in the volumes of hippocampi and amygdalae were not significant (*p* > 0.05). The presence of diabetes mellitus showed only a trend association with lower MoCA scores: 21 ± 5 vs. 27 ± 3, (*p* = 0.06). HD patients with arteriovenous fistula had lower brain oxygenation levels compared to patients with a central venous catheter: 54 ± 13 vs. 63 ± 7, (*p* = 0.02). We did not find a statistically significant difference in oxygenation between the frontal lobe on the side of the AVF and the contralateral side. Patients with an AVF had a longer duration of hemodialysis compared to patients with a permcath (*p* < 0.001; patients with AVF = 63.6 ± 50.2 months, patients with permcath = 21.3 ± 24.3 months).

### Correlation analysis

Significant relations with MRI findings were found only for MoCA but not for rSO_2_. MoCA was higher in HD patients with bigger volumes of amygdala, hippocampus, cortical grey matter, white matter and total brain volume, while MoCA was inversely related to age and the volume of WMH (see Table 4 for details).

**Figure 1:**
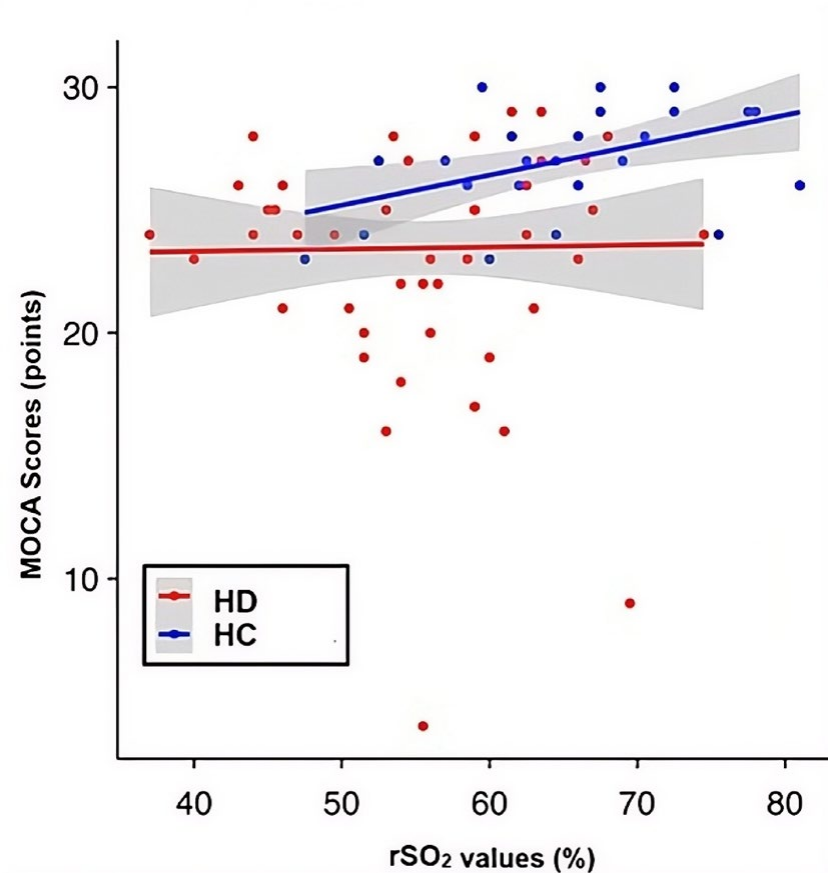
Correlation analysis between rSO_2_ and MoCA values; separately for HD patients and healthy controls rSO_2_ was significantly related to MoCA in the healthy controls (blue), but not in hemodialysis patients (red). Red line: Shows the trend between rSO₂ and MoCA scores in hemodialysis (HD) group. Blue line: Shows the trend between rSO₂ and MoCA scores in the healthy control (HC) group. Gray shaded areas: Represent the uncertainty around each line, showing where the true trend is likely to lie (with 95% confidence). HD–hemodialysis patients; HC–healthy controls; MoCA–Montreal Cognitive Assessment; rSO_2_–regional cerebral oxygen saturation.

Higher brain oxygenation was significantly associated with higher MoCA only in the control group (rho = 0.411, 95% CI [0.020; 0.704], *p* = 0.046), while the relationship was not significant in HD patients (rho = 0.061, 95% CI [−0.352; 0.240], *p* = 0.696) (shown in Figure 1).

A sensitivity analysis was performed by repeating the correlation analysis between MoCA scores and rSO₂ after excluding outlier values. The results remained consistent with the primary analysis — the statistical significance of the associations did not change, and the null hypothesis was again rejected at significance level. The results of this comparison: all data: control (*r* = 0.42, *p* = 0.041), CKD (*r* = −0.061, *p* = 0.6925). Without outliers: control (*r* = 0.42, *p* = 0.041), CKD (*r* = 0.115, *p* = 0.4794).

The number of microbleeds was associated with lower MoCA scores (*ρ* = −0.37, *p* = 0.01). No significant associations were found between MoCA scores, rSO₂ and tested variables, including age, gender, dialysis vintage or hemoglobin levels.

## Discussion

Our study proved that the HD patients had notable brain morphological changes for most of the assessed parameters, worse cognitive functions and lower oxygenation of the frontal lobes than the healthy controls. While cerebral oxygenation was affected only by the presence of AVF, cognitive performance was related to age and to all assessed MRI parameters except lacunar infarcts.

The HD patients had significantly lower brain oxygenation in line with other studies (21, 36–38). We found no significant associations between rSO₂ values and cognitive performance or brain morphological changes in this study. While reduced rSO₂ is a notable finding in HD patients, it may not be the key driver of structural and functional brain changes. The potential effect of cerebral hypoxia may be masked by other pathophysiological mechanisms associated with ESKD, particularly the accumulation of uremic toxins, which have direct neurotoxic effects and contribute to increased neuroinflammation and cognitive decline (39).

This lack of association could also be due to the limitations of the rSO₂ measurement, as the INVOS device measures oxygenation only in the superficial frontal lobes, which may not reflect overall brain oxygenation. Additionally, inter-individual variability in brain oxygenation (as shown in Figure 1) and uneven oxygen distribution between gray and white matter regions of the brain might obscure potential associations. Moreover, we did not measure dynamic changes in oxygenation, particularly during dialysis sessions, when transient hypoxic episodes could occur (40) and brain water content may increase (41).

Patients hemodialyzed via an arteriovenous fistula displayed lower rSO₂ in our study. This may be related to the longer duration of hemodialysis in AVF patients and the potential detrimental effects of chronic dialysis exposure on the brain.

As expected, our study found that the HD patients had worse cognitive performance, which is consistent with a recent meta-analysis (42). The presence of comorbidities may also contribute, as cognitive impairment in hemodialysis patients is likely part of the broader pathophysiological process of the disease. Additionally, a higher proportion of HD patients had a history of smoking, which may have further worsened cognitive outcomes due to its known negative effects on brain function. We also observed a significant correlation between advancing age and cognitive decline among the HD patients. Drew D. A., et al. found that the older the age, the steeper the cognitive decline per year (43).

The HD patients also had a greater burden of WMH and prevalent microbleeds, as well as lacunar infarcts. This may be explained by the higher prevalence of cerebrovascular disease among CKD patients (44). We found that microbleeds and white matter damage were associated with worse cognitive performance. Small vessel cerebrovascular disease that includes microbleeds, lacunar infarcts and white matter changes, is the leading cause of vascular dementia (45).

The HD patients displayed smaller volumes for the whole brain, cortical grey matter, white matter. This is consistent with previous studies (46–48). Brain atrophy is a potential mechanism contributing to cognitive impairment in HD patients, as we found a positive correlation between the aforementioned morphometric parameters and MoCA score. A previous study reported hippocampal atrophy in HD patients (49), which was not evident in our study. The exact reasons for this discrepancy are unclear, but it may be related to the shorter duration of HD vintage in our study (40.5 ± 58.0 months) opposed to the study by Chen H. J. et al. (70.8 ± 53.6 months) (48).

Some, but not all (50) recent studies report atrophy only of the right amygdala (48, 51) or of both amygdalae (52). In our study, it was the left amygdala that was slightly, but significantly smaller in the HD patients. We do not have a clear explanation for this discrepancy. In the study by Lijdsman S. et al. (50), amygdalar sizes were normal. Although the differences are not easy to explain, in the Lijdsman study (median 18.5 years), the patients were considerably younger than ours (median 72.5 years).

The lack of association between rSO₂ and cognitive or structural brain parameters in our study suggests that reduced frontal cerebral oxygenation, measured by NIRS, may not be the dominant mechanism underlying cognitive decline in HD patients. Other factors, such as uremic toxins (12), transient intradialytic hypoxia (40), and changes in brain water content (41), have been proposed as potential contributors in the literature and warrant further investigation in future studies.

The strength of our study is its use of a combined approach with advanced brain MRI techniques, cerebral oximetry and cognitive assessments. This offers new insights that have not been previously explored together. However, several limitations should be acknowledged, including the single-center study design, the cross-sectional design of the study and smaller sample size. Due to the limited sample size, formal multivariable adjustment for all potential confounders was not performed. Additionally, the hemodialysis group had a higher burden of comorbidities, which may have further contributed to the observed cognitive differences. Cognitive performance was evaluated only with a screening method – MoCA. In this study, no in-depth examination of the cognitive functions was performed. The more frequent use of anxiolytic and antidepressant medications in the hemodialysis group may also have influenced the MoCA scores. Estimation of brain oxygenation by rSO_2_ is limited only to the frontal area and this method by principle does not reflect deeper vascular changes, such as in the white matter. Additionally, the exact time of rSO₂ measurements was not systematically recorded. As diurnal variation in cerebral oxygenation may represent a potential confounder, this could not be adjusted for in our analysis. Moreover, we did not collect data on socioeconomic status or education level, factors which can influence cognitive outcomes.

In conclusion, the hemodialysis patients in our study had lower regional brain oxygenation, poorer cognitive functions and evident brain morphological changes in comparison to the healthy controls. However, reduced frontal cerebral oxygenation measured by NIRS was not associated with cognitive decline or brain structural changes, which may reflect limitations of the measurement method rather than the absence of a relationship.

## Data Availability

The raw data supporting the conclusions of this article will be made available by the authors, without undue reservation.
